# High-frequency multimodal atomic force microscopy

**DOI:** 10.3762/bjnano.5.255

**Published:** 2014-12-22

**Authors:** Adrian P Nievergelt, Jonathan D Adams, Pascal D Odermatt, Georg E Fantner

**Affiliations:** 1 Laboratory for Bio- and Nano-Instrumentation, École Polytechnique Fédérale de Lausanne, Batiment BM 3109 Station 17, 1015 Lausanne, Switzerland

**Keywords:** atomic force microscopy, multifrequency imaging, nanomechanical characterization, photothermal excitation, small cantilevers

## Abstract

Multifrequency atomic force microscopy imaging has been recently demonstrated as a powerful technique for quickly obtaining information about the mechanical properties of a sample. Combining this development with recent gains in imaging speed through small cantilevers holds the promise of a convenient, high-speed method for obtaining nanoscale topography as well as mechanical properties. Nevertheless, instrument bandwidth limitations on cantilever excitation and readout have restricted the ability of multifrequency techniques to fully benefit from small cantilevers. We present an approach for cantilever excitation and deflection readout with a bandwidth of 20 MHz, enabling multifrequency techniques extended beyond 2 MHz for obtaining materials contrast in liquid and air, as well as soft imaging of delicate biological samples.

## Introduction

The atomic force microscope (AFM) has developed into an extremely useful and versatile tool for nanometre-scale visualization and mechanical characterization. In recent years, several methods have been developed for simultaneous measurement of topographical and mechanical information by using AFM, opening up new possibilities for biology and materials science [1–9]. A key enabling trend in the technological development of AFM has been the drive to minimize the cantilever size and maximize the resonance frequency, while maintaining acceptable spring constants [10–12]. Increasing the cantilever resonance frequency enables faster imaging and force spectroscopy [12–17], and small, high-frequency AFM cantilevers have less viscous drag, lowering force noise [18]. Many of the techniques for extracting mechanical information during imaging utilize higher cantilever resonant modes. Here, the ability to detect cantilever motion at high-frequencies becomes an increasingly critical requirement that is often beyond current instrument capabilities.

In addition to the availability of small, high-frequency cantilever probes and optical beam deflection (OBD) systems with a sufficiently small focus spot to use small cantilevers [12,19], two key practical aspects have limited the widespread use of AFM imaging at frequencies beyond 2 MHz: cantilever drive and deflection readout. In liquids, traditional piezo-based cantilever excitation leads to the generation of numerous system resonances that can mask or fail to drive the desired cantilever resonances and complicate subsequent interpretation and analysis. This problem is accentuated at high frequencies. Alternately, the cantilever can be directly driven by using techniques including magnetic [20], resistive thermal [21], integrated piezotransducer [22] or photothermal [23–24] excitation, eliminating this effect. Of the direct drive techniques, photothermal-based excitation has the benefit that it is compatible with most standard AFM cantilevers and, although long-established, has recently gained renewed interest [25–30]. Although the efficiency of photothermal excitation varies with different coatings, even uncoated cantilevers have been shown to work [31]. Furthermore, photodiode readout electronics in the OBD system typically have been restricted to approx. 2 MHz for standard systems and a maximum of 10 MHz for highly-optimized systems [27]. Even for cantilevers with fundamental resonances of 1–2 MHz, at the second or higher modes this limit is quickly reached. Only a small number of alternative approaches for moving past this limitation have been explored; these include heterodyne optical beam and interferometric detection [32–34] and current-based translinear readout circuitry [35]. Of these approaches, the latter shows excellent potential for low-noise and high-bandwidth direct OBD readout.

Surmounting these technological challenges has thus far remained the domain of a handful of highly-specialized instruments. In this report, we present high-resonance-frequency bimodal AFM imaging by using an AFM readout head designed for high-frequency drive and readout of small cantilevers. Our head is compatible with the Bruker MultiMode AFM, a widely used commercial system. We show that our system has the ability to stably drive small AFM cantilevers in both air and fluid at in bandwidth exceeding 20 MHz, with a detection noise floor comparable to lower bandwidth commercial systems. We demonstrate the application of our instrument towards multifrequency materials contrast imaging of a polymer blend in both air and fluid, and gentle, high-resolution imaging of an F-actin fibre in fluid.

## Results and Discussion

### Instrument design

**Figure 1:**
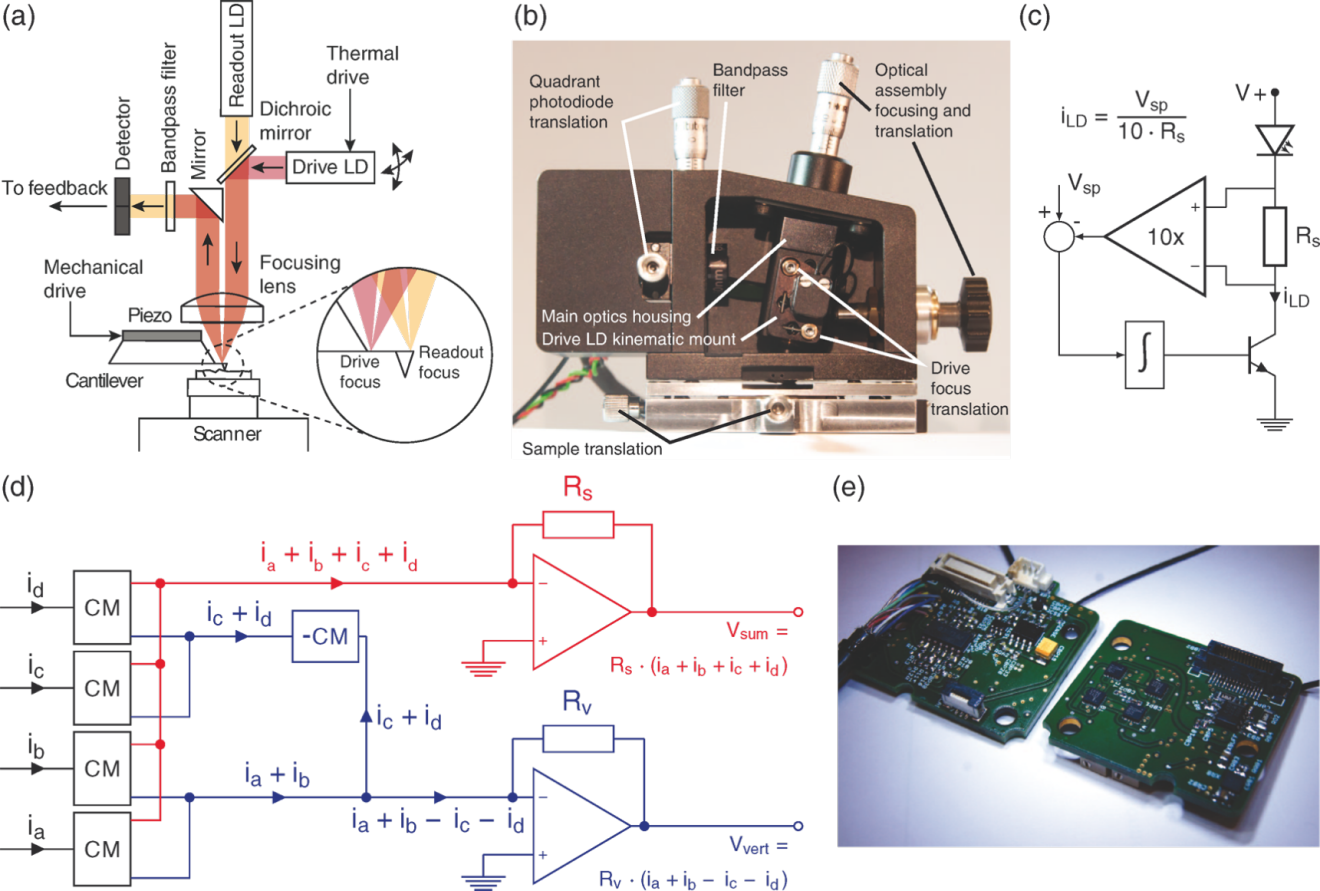
a) Schematic of the optical drive and detection setup. The drive laser focus can be positioned relative to the readout laser focus through an adjustable kinematic mount. b) Photograph of the assembled readout head. The head can be mounted directly onto Bruker MultiMode scanners. c) Schematic of the constant current driver circuit for the photothermal drive laser. d) Simplified functional schematic of the high-bandwidth readout electronics. Transistor-based current arithmetic greatly improves bandwidth and reduces noise. Only the sum and vertical channels are shown for clarity; the horizontal deflection is also calculated. (CM = current mirror, −CM = current subtractor). e) Photograph of the readout electronics circuitry. One circuit board provides power conditioning and the drive laser control, the second board calculates the readout arithmetic.

The basis for our optical design is a modular AFM readout head design we have reported earlier [36]. The modular nature of this head permits the easy exchange of the optical assembly, allowing for the integration of custom optics elements such as a photothermal drive. Figure 1a illustrates the architecture of our photothermal optical assembly, and Figure 1b shows a picture of the optical and head assembly. The optical design uses a spatial separation approach to separate the incident and reflected light paths, with the additional photothermal drive laser mounted onto the core optics block via an adjustable kinematic mount (see section “Optical beam deflection setup”). Since the drive laser diode has to be modulated at frequencies beyond the capability of most commercial drivers, we used a custom-built wideband constant-current source (Figure 1c). Adjustment of the incidence angle of the collimated drive beam onto the focusing lens translates the focal position. This architecture permits the relative position of the two laser spots to stay fixed when the lasers are aligned to the cantilever. Furthermore, because the optical axis of the assembly is normal to the cantilever top surface, we eliminate the need for refocusing when positioning the foci on the cantilever.

Voltage-based arithmetic, which is used by most quadrant photodiode readouts, uses operational amplifiers to calculate the vertical and horizontal deflections of the laser spot. Our readout in contrast uses translinear loops, allowing us to calculate both deflections in currents as shown by Enning et al. [35]. Figure 1d shows a conceptual schematic of the readout circuit. The photodiode currents are first copied with current mirrors. The currents are then added or subtracted as necessary to generate the sum, vertical and horizontal signals as currents. Finally, transimpedance amplifiers convert the current signals into voltages. The use of current mirror based readout has two major advantages over a conventional, purely operational amplifier based readout. The large increase in speed is achieved by the very low input impedance of current mirrors, thus countering the negative impact of diode parasitics on the total bandwidth. Additionally, the serial nature of the inherently slow voltage-based addition–substraction–division circuits poses a significant bandwidth limitation to voltage-based readouts, which can be circumvented by using current-based arithmetic, which is only limited by an inherently fast transimpedance stage. Besides the increased bandwidth, as operational amplifiers are very complex many-transistor devices, the effective reduction in the total number of transistors used has the potential to allow for a very low electronic noise floor.

### Characterization

**Figure 2:**
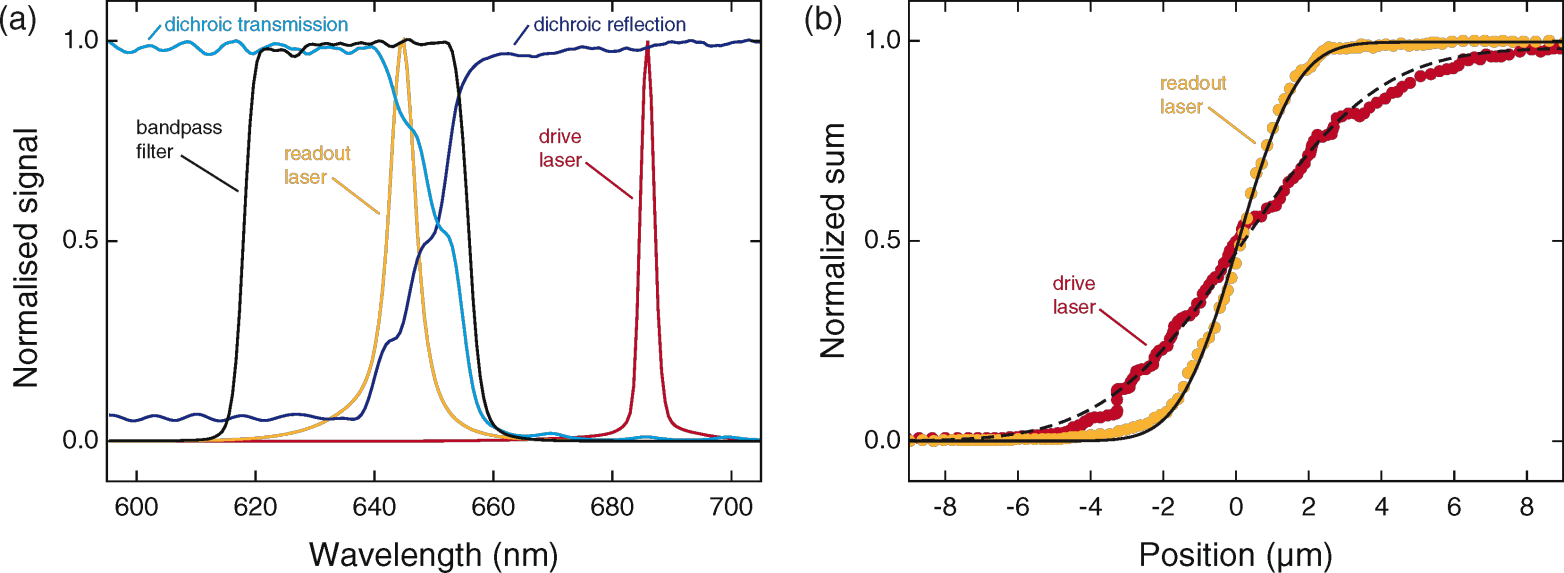
a) Measured spectra of the major optical components in the readout design. b) Measurement of the beam waist of the readout and drive laser. The 1/*e*^2^ waist of the readout and drive laser are 2.6 μm and 5.9 μm, respectively.

We characterized the performance of the major optical components in our optical design by using a spectrometer (9405CB, Hamamatsu, Hamamatsu City, Japan). Figure 2a presents the normalised spectra for the two lasers, the bandpass filter and the dichroic mirror. The peak emission wavelengths of the readout and drive lasers were measured at 645 nm and 686 nm, respectively. The readout laser sits well within the pass-band of the bandpass filter, measured at 618 nm to 656 nm at 50% transmission. At the drive laser emission wavelength we measured an extinction higher than OD3 from the bandpass filter, effectively reducing cross-talk from the drive laser to below 0.03% at typical optical powers used during imaging (approx. 1 mW for the readout and approx. 0.2 mW for the AC component of the drive). We measured that the dichroic mirror has only 80% transmission at the readout laser wavelength. While the readout laser power can be adjusted to maintain sufficient intensity at the photodiode, this clipping introduces some additional stray light in the system. We chose the dichroic mirror primarily for cost reasons, and expect that a minor performance increase could be obtained by choosing a dichroic mirror with a tailored stop-band transition.

We measured the beam waist of the readout and drive lasers by using a modified knife-edge technique. An interferometer (NA, SIOS Meßtechnik, Ilmenau, Germany) tracked the position of the optics block as it was swept across a cantilever, and the sum signal from the photodiode was recorded. We inferred the spatial position of the focal spot relative to the optics block geometrically. An error function fit to the data yielded a beam waist measurement of 2.6 μm for the readout laser and 5.9 μm for the drive laser (Figure 2b). In contrast to other implementations [27,29–30], our choice of two closely-spaced laser wavelengths simplifies the simultaneous focusing of the two laser spots. For these beam waists, we calculate Rayleigh lengths of 33 μm and 160 μm, well within the estimated 13 μm chromatic focal shift of our optical system obtained by using Zemax 13 (Radiant Zemax LLC, Redmond, WA, USA).

**Figure 3:**
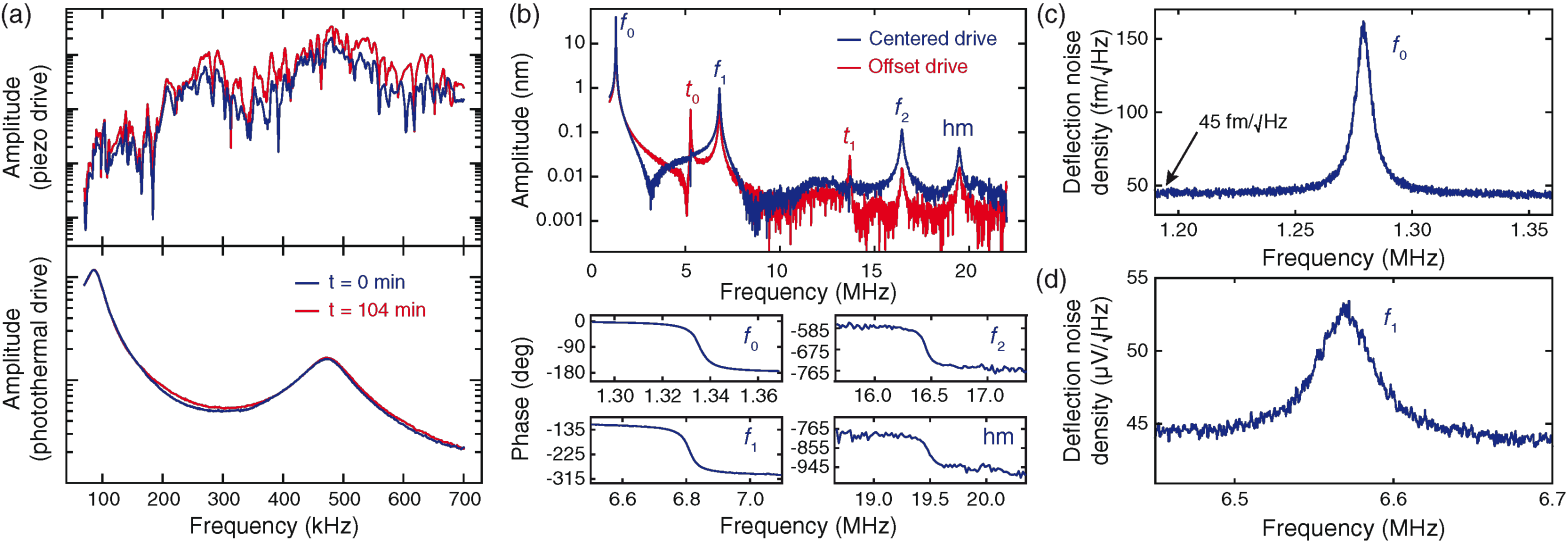
Cantilever drive and deflection readout characterization. a) In contrast to piezo excitation (top curves), photothermal excitation (lower curves) cleanly and consistently drives the first two resonances for more than 100 min. b) The photothermal tunes show resonances up to 19.5 MHz, demonstrating the wide bandwidth with clean phase responses for selected modes. By offsetting the drive laser laterally on a triangular cantilever (Bruker FastScan C), torsional resonances can be excited (red curve). Visible are the first three flexural modes (*f*_0_, *f*_1_ and *f*_2_), the first two torsional modes (*t*_1_ and *t*_2_), and a complex higher resonant mode (hm). c) Thermal noise peak of the first flexural mode of a FastScan A cantilever, with a baseline noise floor of 45 fm/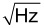. d) Thermal noise peak of the second flexural mode of a FastScan A at 6.6 MHz.

While piezo-driven tapping mode imaging in liquid is used extensively in the literature, the strong dependence of the excitation efficiency on the geometry around the cantilever makes driving high-resonance-frequency cantilevers difficult or impossible. Changes in the surrounding liquid, which conducts acoustic energy from the piezo into the surrounding structures, can drastically alter the cantilever drive efficiency. These effects also make long term imaging difficult and hard to control. Localized excitation techniques such as photothermal excitation cause negligible ambient vibrations, therefore the excitation efficiency does not depend on the total liquid volume surrounding the cantilever and generally yields a much cleaner drive. Figure 3a illustrates this effect. A FastScan C cantilever (Bruker AFM Probes, Camarillo, CA, USA) was placed in a hanging water droplet and alternately driven with photothermal and piezo excitation. The first two resonant modes are clearly visible in the photothermally-driven amplitude signal, whereas they are hidden within the “forest of peaks” [37] in the piezo-driven amplitude signal. As the droplet dried over a period of approx. 100 min, the piezoelectric tune changed significantly, while the photothermal tune shows nearly no variation. In particular, the second resonance excitation (Figure 3a) increases by 50% to 100% under piezo excitation, but by only 3% under photothermal excitation.

We measured the ability of our system to drive and detect multiple cantilever eigenmodes at the corresponding high frequencies by using a FastScan A cantilever (Bruker AFM probes). Figure 3b shows the driven response of the cantilever with clearly detected resonant modes up to 20 MHz (blue curve). The first three flexural modes, the first two torsional modes, and a complex higher mode are visible. The lower part of Figure 3b shows clear phase shifts of 180° through each of the first three flexural resonances and the complex higher mode. Translating the drive laser focus spot can preferentially excite different resonant modes of the cantilever. We enhanced the excitation of the first two torsional modes by approximately one order of magnitude by placing the drive laser spot offset from the middle of the cantilever (red curve in Figure 3b). We confirmed our identification of the resonant modes by using a finite element model of the cantilever (Comsol 4.3b, Comsol, Inc., Burlington, MA, USA).

By using the thermal tune method and a FastScan A cantilever in air, we measured a baseline noise level of 45 fm/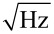 for our deflection readout. Figure 3c shows the thermal noise peak of the first flexural mode, while Figure 3d shows the thermal noise peak of the second flexural mode. We expect that further optimization of our system for noise performance will decrease the baseline noise value further [35].

### Dissipation imaging

#### Bimodal imaging

The capability for clean, high-frequency cantilever excitation, and low-noise, high-frequency deflection readout provide a powerful platform for extending multifrequency techniques to higher frequencies. For simultaneous high-frequency imaging and mechanical property mapping, we use a bimodal resonant technique which tracks topography in amplitude modulation on the first eigenmode [5,38]. This mode is one of the possibilities of achieving materials contrast while simultaneously tracking topography. The resonant excitation power needed to keep the second eigenmode at a specific amplitude is mapped, while a phase locked loop (PLL) ensures resonant excitation. Topography feedback deconvolutes material specific effects acting on the second resonance. As the resonant amplification is kept constant with the PLL, the amount of drive signal needed to keep the amplitude constant is proportional to the power dissipated in the tip–sample interaction. The power dissipation (*P*_diss_) is calculated from the applied excitation signal (*V*_ex_·sin (2π*f*)) and the intrinsic power dissipation of the cantilever (*P*_0_) as

[1]
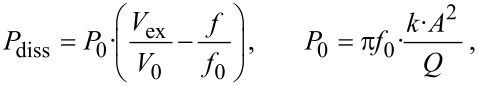


where *V*_0_ is the excitation voltage, *f*_0_ the excitation frequency, *k* the spring constant, *A* the amplitude and *Q* the quality factor far from the surface [39]. The acquired dissipation is, to a first approximation, only dependent on the materials properties and the additional squeeze-film damping of the cantilever, the latter of which is roughly constant while in feedback.

**Figure 4:**
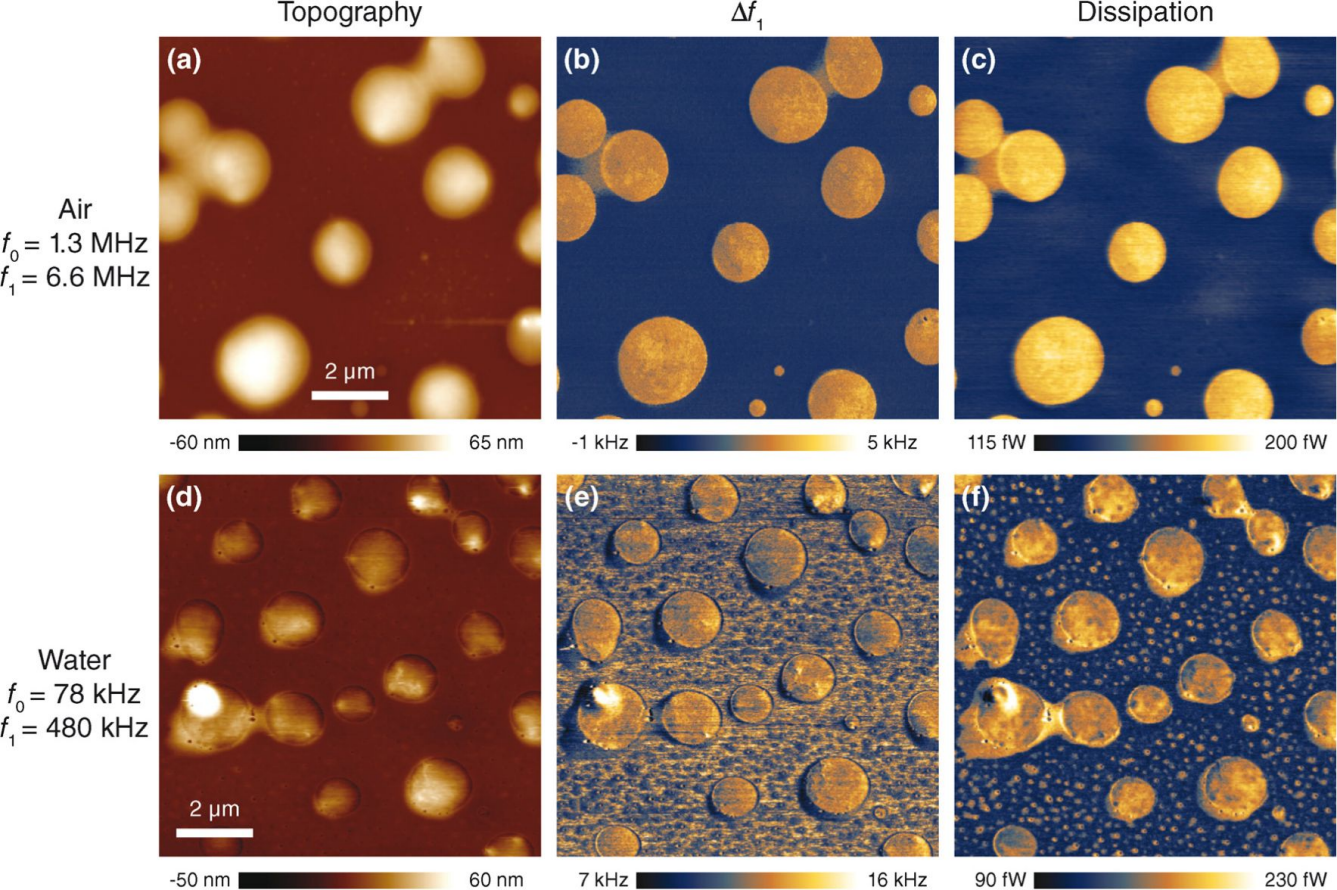
Bimodal AFM imaging of a PS/PMMA polymer blend with small, high-frequency cantilevers in both air (panels a–c) and water (panels d–f). Panels a and c show topography based on amplitude modulation of the fundamental resonance. Panels b and e show the resonance frequency shift of the first higher resonant mode, and panels c and f show the drive amplitude needed to keep the first higher resonant mode at constant amplitude, related to the energy dissipation in the tip–sample interaction.

We used a thin-film blend of polystyrene (PS) and poly(methyl methacrylate) (PMMA) as a sample (PS–PMMA–15M, Bruker AFM probes); its separation into soft and hard domains makes it a widely used standard for materials contrast imaging. For imaging in air, we used a FastScan A cantilever with the fundamental and first higher flexural resonant modes at 1.3 MHz and 6.6 MHz, respectively. Figure 4a shows the resulting topography image, while Figure 4b and Figure 4c show the frequency shift and dissipation images, respectively. A clear difference in dissipative properties of the two phases can be observed, as is expected. For imaging in water, we used a FastScan C cantilever with drive frequencies at 78 kHz and 480 kHz for the fundamental and first higher resonant modes, respectively. Amplitudes of the first eigenmode were set to 8 nm free amplitude with a setpoint of around 50–60% for both air and water imaging. The second eigenmode was set to 54 pm in air and 86 pm in water. Figure 4d–f present the topography, frequency shift and dissipation images, respectively. The dissipation images show a very clear step contrast for the softer globular areas with no visible effects from the topography feedback. At present, we are uncertain of the source of the apparent contrast inversion at the edges of the globular areas in Figure 4d versus Figure 4a, although it may be due to surface restructuring of the polymer blend in water [40].

One issue of note is that higher eigenmodes have an inherently higher dynamic stiffness that can be up to two orders of magnitude larger than the fundamental mode. This can be problematic for softer samples, as the power dissipated into the sample increases linearly with the spring constant according to Equation 1. The increased optical lever sensitivity (OLS) of the second mode helps in being able to use smaller amplitudes, which reduces the power dissipation and, consequently, the damage to the sample. In order to improve the topography tracking, the bandwidth of the first eigenmode should be increased. Moving to smaller cantilevers allows for higher resonance frequencies which improves the detection bandwidth, while at the same time keeping spring constants low. In the case of imaging in a highly damped environment such as water, the bandwidth of the cantilever will increase due to viscous damping, however the detection bandwidth scales linearly with the dissipated power. The linear scaling is due to the fact that both the dissipated power (see Equation 1) and the cantilever AC-bandwidth, which is proportional to (*f*_0_/*Q*), scale proportionally with the resonance frequency and inversely with the quality factor. The increased ratio of resonance frequency to spring constant makes it clear that the use of small cantilevers is ideally suited for low-dissipation imaging on multiple dynamic modes.

#### Drive amplitude modulation imaging

**Figure 5:**
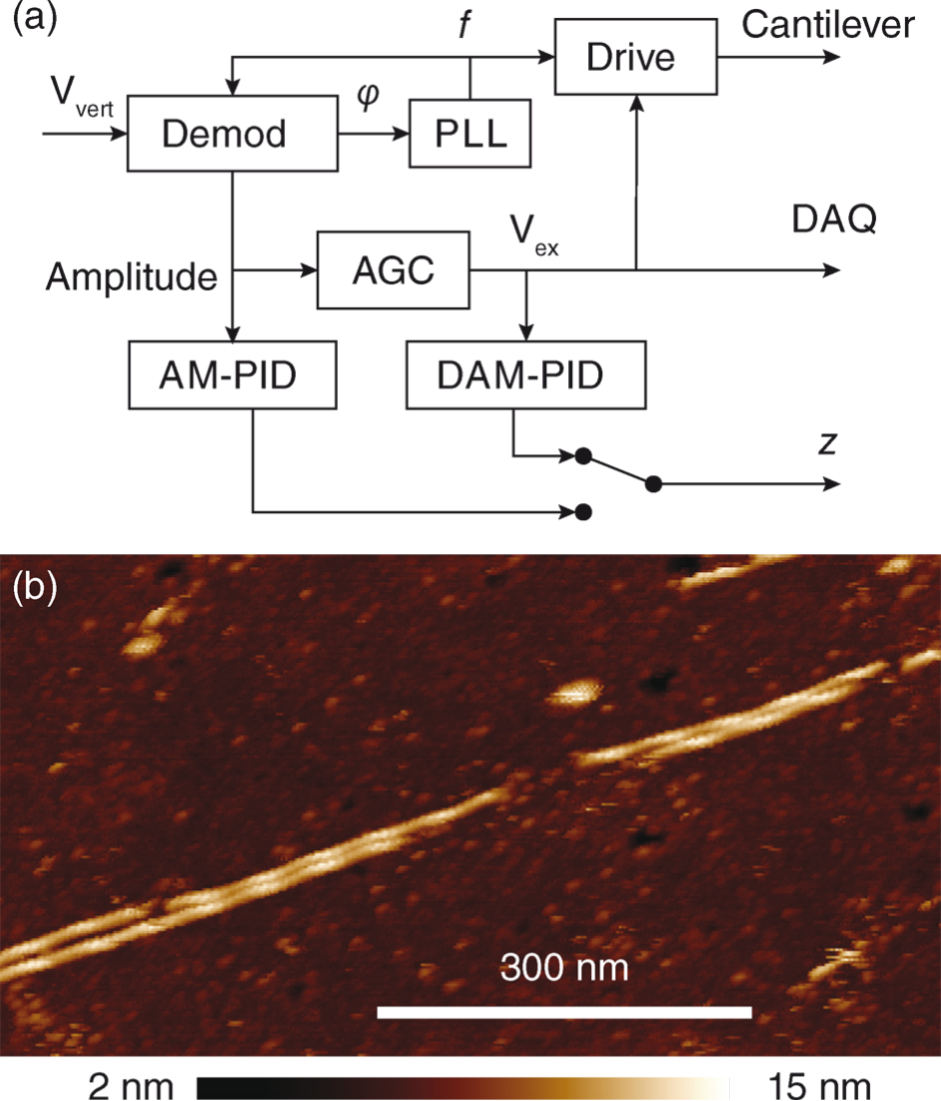
a) Schematic of the drive amplitude modulation feedback compared with standard amplitude modulation imaging. Instead of using the oscillation amplitude as feedback variable like in conventional amplitude modulation mode, the oscillation amplitude is kept constant and the drive amplitude required to keep it constant is used as feedback variable. The drive is then enforced to a setpoint above the free drive, resulting in a stable topography feedback. b) High-resolution DAM imaging in liquid of soft F-actin fibres on (3-aminopropyl)triethoxysilane coated glass. Both the sub- and superstructure of the protein are visible.

For biophysical imaging with atomic force microscopy, the ability to scan delicate samples in high resolution is required when investigating soft nanostructures. A related technique to the dissipation imaging described above, drive amplitude modulation (DAM) is an imaging mode that allows for the control of the dissipation in the AC-mode tip–sample interaction [41]. Figure 5a provides a schematic of the drive amplitude modulation imaging setup. By using a PLL in combination with an automatic gain controller, the amplitude of the first eigenmode of the cantilever is kept at a constant setpoint while the resonance frequency of the mode is tracked. The scanner feedback loop is then closed by enforcing a higher drive amplitude than the free drive amplitude. As the tip–sample distance decreases, the force interaction becomes stronger and energy is lost from the cantilever oscillation. By using this technique, the non-monotonic tip–surface interaction potential is mapped onto a monotonic function. By controlling for a constant energy loss in this way, soft imaging with very small amplitudes down to 100 pm can be realized; however, an unclean cantilever excitation can negatively impact the imaging. Our photothermal readout head provides the capability for a clean drive and thus enables this technique in water. Figure 5b demonstrates gentle imaging of a sample of F-actin fibres deposited on a (3-aminopropyl)triethoxysilane-coated glass surface in liquid. F-actin is a fibre-forming protein that plays a role in the cytoskeleton. F-actin filaments are a notoriously difficult sample for AFM due to their fragility and quick contamination of the cantilever tip. Thus far, successful AFM imaging reports have used either extremely soft cantilevers or hopping-mode imaging methods with very low trigger forces [42–44]. By using our system we were successfully able to take high resolution images of deposited fibres showing the helical structure of the fibre and an underlying substructure related to the individual protein subunits. Even by using a comparatively stiff cantilever for biological imaging (*k* = 0.8 N·m^−1^) with a high resonance frequency in fluid, there was little apparent imaging damage to the structure once the feedback gains were adjusted properly.

## Conclusion

Imaging gently and quickly is a constant challenge in AFM. Small cantilevers are well suited to low-dissipation imaging, especially in multifrequency imaging modes, since the spring constants of higher eigenmodes can be kept at reasonable values without sacrificing imaging bandwidth. However, their application in multifrequency techniques has been restricted due to instrument capability limitations. By using photothermal actuation of small cantilevers along with a current-based deflection readout, we have shown bimodal imaging of a polymer blend in both air and liquid, with amplitudes of the second mode well below a nanometre at previously inaccessible cantilever resonance frequencies. We furthermore demonstrated gentle, low-dissipation imaging of F-actin in drive amplitude modulation mode with oscillation amplitude below 1 nm. We believe that the combination of small cantilevers, clean photothermal actuation and high-frequency, low-noise deflection readout will be of great benefit for multifrequency AFM imaging faster and with less tip–sample dissipation.

## Experimental

### Optical beam deflection setup

In our optical beam deflection system, we combine a collimated 5 mW 637 nm readout laser diode (HL6355MG, Conrad, Dietikon, Switzerland) and a 50 mW 685 nm drive laser diode (HL6750MG, Thorlabs, Newton, NJ, USA) by using a 650 nm short-pass dichroic mirror (69-218, Edmund Optics, Barrington, NJ, USA). The laser diodes are each collimated in individual housings by using an aspheric lens (A390-A, Thorlabs). The readout laser diode is driven from an external commercial laser diode driver (LDX3412, ILX Lightwave, Irvine, CA, USA) and modulated with a custom-built push–pull oscillator circuit (EL6204, Intersil, Milpitas, CA). The incident and reflected beam paths are spatially separated such that they use separate parts of the focussing lens (A390-A, Thorlabs). A right angle mirror (48-383, Edmund Optics) redirects the reflected laser beams towards a quadrant photodiode (S4349, Hamamatsu, Hamamatsu City, Japan). A 625 nm centre wavelength 50 nm bandpass filter (86-941, Edmund Optics) blocks the drive laser beam from the quadrant photodiode. 0.20 mm pitch adjustment screws (F2D5ES10, Thorlabs) permit translation of the drive laser focal spot with approximately 0.34 μm and 0.18 μm per degree of screw rotation along the cantilever length and width, respectively.

### Actin filament preparation

A 12 mm diameter glass coverslip (Novoglas Labortechnik) was cleaned with piranha solution (1:3 ratio of hydrogen peroxide to sulphuric acid), rinsed with distilled water and dried by a nitrogen stream. The coverslip was then immersed in a solution of (3-aminopropyl)triethoxysilane (0.5% in water) (A3648, Sigma-Aldrich, St. Louis, MO, USA) for 10 min then rinsed. The coverslip was then dried in an oven for approximately one hour at 65 °C in a vertical position and subsequently glued onto a steel disc for imaging. F-actin was prepared according to the protocol of the manufacturer (BK003, Cytoskeleton, Inc., Denver, CO, USA). An amount of 1 μL F-actin was stabilized with 3 μL Alexa Fluor 488 Phalloidin (A12379, Life Technologies, Carlsbad, CA, USA) and diluted to a final volume of 40 μL in buffer (2 mM MgCl_2_, 1 mM EGTA, 20 mM imidazole·HCl, pH 7.6). Of this, 10 μL was deposited onto the coverslip and incubated for one minute before more buffer was added onto the sample prior to imaging.

### Dissipation imaging setup

The imaging modes as described in section “Dissipation imaging” were implemented by using a commercial controller (Nanoscope 5, Bruker) in combination with a digital high-frequency multifunction instrument (UHFLI, Zurich Instruments, Zurich, Switzerland), interfaced via a signal access module (SAM III, Bruker). The scan generation and data acquisition is handled by the AFM controller while feedback and PLL are provided by the multifunction instrument. A custom-built wideband up/down-scaling amplifier provides voltage level compatibility between the two components. The vertical signal from the detector is accessed directly from the detector via a 50 Ω coax cable and wired to the downscaler and the AFM controller. A external high-voltage amplifier, identical to the one in the Nanoscope 5 controller, is driven off the multifunction instrument to displace the piezo tube in the *z*-direction.

The dissipation images are calibrated by Equation 1. Since the amplitude of the second eigenmode is difficult to calibrate by approach curves due to the motion of the first eigenmode, we estimate the difference in sensitivity from eigenmode calculations using finite element analysis. We find the ratio of the second eigenmode OLS with respect to the first eigenmode OLS to be a factor 5.85 and 6.0 for the FastScan A and FastScan C cantilevers, respectively. The spring constants of the two cantilevers are calibrated by using the thermal noise method. We measure *k*_0_ = 15.4 N·m^−1^, *k*_1_ = 470 N·m^−1^ for the first and second eigenmode of the used FastScan A and *k*_0_ = 0.85 N·m^−1^, *k*_1_ = 94 N·m^−1^ analogous for the FastScan C cantilever. We calculate *P*_0_ = 176 fW for a 54 pm amplitude in air with the FastScan A and *P*_0_ = 150 fW for a 86 pm amplitude in water with the FastScan C. Stock coatings were used (approx. 100 nm Al for the FastScan A and approx. 60 nm Ti/Au for the FastScan C).
